# Exacerbation of Paraneoplastic Neurologic Syndrome After Immune Checkpoint Inhibitor Treatment in Advanced Small Cell Lung Cancer: A Case Report

**DOI:** 10.7759/cureus.79272

**Published:** 2025-02-19

**Authors:** Toru Hamada, Koji Inoue, Masashi Watanabe, Norihiko Nakanishi

**Affiliations:** 1 Department of Respiratory Medicine, Ehime Prefectural Central Hospital, Matsuyama city, Ehime, JPN; 2 Department of Neurology, Ehime Prefectural Central Hospital, Matsuyama city, Ehime, JPN

**Keywords:** antineural antibodies, atezolizumab, immune checkpoint inhibitors, paraneoplastic neurologic syndrome, small-cell lung cancer

## Abstract

Paraneoplastic neurologic syndrome (PNS) is an association of various cancer types and is reported to occur in small-cell lung cancers (SCLC). It is a nervous disturbance caused by immune-mediated mechanisms with various neurological symptoms. Immune checkpoint inhibitors (ICIs) are increasingly being used to treat SCLC.

We report the case of a 69-year-old woman with SCLC complicated by PNS who experienced worsening neurological symptoms (Sensory disturbance, sensory ataxia, and autonomic symptoms) after receiving atezolizumab. The patient’s neurological symptoms partially improved after the immunological treatment. Her neurological symptoms did not worsen and her lung cancer remained stable.

We believe that ICIs will be increasingly used in the future for SCLC and that we will encounter more cases of PNS that may merge with SCLC deteriorating after receiving ICIs. We describe one case and review the clinical characteristics of patients with SCLC whose PNS was exacerbated by ICIs, including patients with SCLC whose PNS were present prior to the initiation of ICIs. We suggest that neurological symptoms suggestive of PNS should be carefully monitored after the introduction of ICIs for SCLC.

## Introduction

A combination of cytotoxic anticancer agents and immune checkpoint inhibitors (ICIs) is commonly used to treat advanced small-cell lung cancers (SCLC). ICIs have been shown to contribute to the exacerbation of pre-existing autoimmune diseases in patients [1]. Paraneoplastic neurologic syndrome (PNS) is a complication of SCLC and is thought to present with neuropathy due to autoimmune mechanisms. There are no clear reports on the age at which PNS tends to occur in SCLC patients. However, it has been reported that PNS in SCLC tended to occur more frequently in males [2,3].

A previous study reported that the prevalence of autoimmune diseases among lung cancer patients ranges from 14 to 25% [4]. In addition, another report revealed that 21% of patients experienced exacerbation of autoimmune diseases following the administration of ICIs [5]. The paraneoplastic neurological syndrome manifests in 1-9% of cases of SCLC [2,3], though the precise incidence remains uncertain. The severity of neuropathy can range from mild to severe manifestations.

The clinical characteristics of patients with PNS associated with SCLC and exacerbated by ICIs are not well described. We encountered a case of worsening neurological symptoms in a 69-year-old woman with PNS associated with SCLC treated with an ICI. In addition to describing this case, we review the clinical characteristics of patients with SCLC whose PNS was exacerbated by ICIs, including patients with SCLC whose paraneoplastic neurological symptoms were present prior to the initiation of ICIs [6].

## Case presentation

A 69-year-old woman was referred to our hospital for the evaluation of general fatigue, anorexia, and a cough that had persisted for over a month. She had a history of hypertension and was taking benidipine 4 mg. She had a 47-pack-year smoking history. Blood tests revealed hyponatremia (Serum Na 122mEq/L) and a high level of tumor marker (pro-gastrin releasing peptide (GRP) 3666 pg/mL). Blood tests revealed hyponatremia (Serum Na 122mEq/L) and elevated levels of a tumor marker (pro-GRP 3666 pg/mL). Based on the results of positron emission tomography-computed tomography (PET-CT), brain magnetic resonance imaging (MRI), and bronchoscopy, she was diagnosed with left upper lobe extensive SCLC (cT4N3M1b, LYM in the 8th TNM classification). At the time of definitive diagnosis, she did not complain of sensory disturbance in the distal extremities or dizziness.

In view of her performance status (PS) that was based on the Eastern Cooperative Oncology Group was 3, chemotherapy with carboplatin and etoposide without ICI was initiated. When she was diagnosed with SCLC, the largest short diameter of the mediastinal lymph nodes was 48.5 mm, and that of the left axillary lymph nodes was 16.4 mm. Her lung cancer and lymph node metastases rapidly shrank; the largest short diameter of the mediastinal lymph nodes was 8.7 mm, and the left axillary lymph nodes could no longer be detected after two courses of carboplatin and etoposide. In addition, her fatigue and anorexia improved. However, she had noticed a sensory disturbance (numbness) in her distal extremities, constipation, and walking instability, which did not interfere with her daily life around the second course of that chemotherapy (two months after the initiation of the treatment). As her PS improved to 1, the ICI atezolizumab was added in the third course.

After completing the fourth course of treatment (three months after her diagnosis), maintenance therapy with atezolizumab 1200 mg per three weeks was initiated. Her sensory disturbance in the distal extremities and walking instability gradually worsened, and orthostatic hypotension and constipation continued. After two courses of atezolizumab maintenance therapy, her PS dropped to 3 due to worsening sensory disturbance in the distal extremities and walking instability; therefore, maintenance therapy with atezolizumab was discontinued, and she was treated with symptomatic medication: duloxetine 20 mg, mecobalamin 1500 μg, and pregabalin 150 mg. Her symptoms did not improve, and she was referred to the Neurology department. She presented with general fatigue, decreased motivation, and depressed mood. Neurological examination revealed orthostatic hypotension, sensory disturbances in the distal extremities (numbness in the region distal to both wrist and knee joints), decreased deep tendon reflexes, and ataxia. Her blood test revealed the following parameters: white blood cells 11430/μL, red blood cells 437×10^4^/μL, platelets 52.5×10^4^/μL, CRP 0.14 mg/dL, vitamin B1 28.3ng/mL (normal range 21.3-81.9ng/mL), vitamin B12 1617pg/mL (normal range 189-914 pg/mL), folic acid 7.8 ng/mL (normal value >4.0 ng/mL), negative for antinuclear antibodies, negative for anti-SS-A antibodies, negative for anti-U1RNP antibodies, negative for myeloperoxidase-specific antineutrophil cytoplasmic antibody (MPO-ANCA), and negative for PR3-ANCA. No obvious abnormalities were observed on head and neck CT. There were also no obvious abnormalities on the head MRI. She did not consent to cerebrospinal fluid examination. In the nerve conduction test, the compound muscle action potential (CMAP) and sensory nerve action potential (SNAP) amplitudes were reduced in the median nerve, and the conduction velocity was at the lower limit of normal. The ulnar nerve had a normal CMAP amplitude and conduction velocity; however, the SNAP amplitude was reduced. The CMAP amplitude of the tibial nerve was reduced; however, the conduction velocity was within the normal lower limit. The SNAP amplitude of the peroneal nerve and conduction velocity were severely reduced. The CMAP amplitudes were normal. She was treated for SCLC and was diagnosed with a variety of neurological disorders in addition to numbness in the extremities. Due to the variety of symptoms, it was thought that the cause was paraneoplastic neuropathy rather than drug-induced neuropathy caused by carboplatin and etoposide, so serum anti-nerve antibodies were submitted, and anti-Hu, anti-AMPH, anti-Ri, anti-SOX-1, and anti-Zic4 were positive. When using the PNS Care Score (high-risk phenotype (SSN) 3 points), (high-risk antibody (anti-Hu antibody) 3 points), and (SCLC 4 points) for a total of 10 points, the diagnostic level was “Definite”. Therefore, she was diagnosed with PNS [7].

We checked the thyroid function test that was performed before the introduction of carboplatin. Thyroid-stimulating hormone (TSH) was 2.75 μIU/mL (normal range: 0.35-4.94), FT3 was <1.5 pg/mL (normal range: 1.88-3.18), and FT4 was 0.88 ng/dL (normal range: 0.7-1.48), with only FT3 being low value, and it was judged to be a case of hypothyroidism due to malnutrition.

Two months after starting atezolizumab, thyroid function was checked. An increase in FT3 and thyroid hormones were observed, with TSH <0.03μIU/mL (normal range: 0.35-4.94), FT3 2.06pg/mL (normal range: 1.88-3.18), FT4 8.2ng/dL (normal range: 0.7-1. 48), and FT3 and thyroid hormone levels increased. Anti-TSH receptor antibodies, anti-thyroglobulin antibodies, and anti-thyroid peroxidase antibodies were negative. On thyroid ultrasound, the echogenicity of the thyroid parenchyma was heterogeneous, and there was no increase in blood flow. There were no symptoms associated with hyperthyroidism, such as tachycardia or sweating; therefore, the patient was placed under observation. One month later, she was experiencing depressed mood, and a blood test was performed to check thyroid-related items, which showed a decrease in thyroid hormone levels and an increase in TSH, with TSH 19.6 μIU/mL (normal range: 0.35-4.94), FT3 <1.5 pg/mL (normal range: 1.88-3.18), and FT4 0.79 ng/dL (normal range: 0.7-1.48), which was consistent with the course of destructive thyroiditis. (normal range: 0.7-1.48), and there was a decrease in thyroid hormone levels and an increase in TSH levels, which was consistent with the course of destructive thyroiditis. As she showed no signs of infection and developed destructive thyroiditis after starting atezolizumab, the patient was diagnosed with atezolizumab-induced destructive thyroiditis.

She was diagnosed with destructive thyroiditis due to immune-related adverse events (irAEs) from atezolizumab before being introduced to the Neurology department and treated with levothyroxine. This led us to consider neuropathy due to irAEs in addition to tumor-associated neurological syndrome as the cause of her neurological symptoms. The patient received intravenous methylprednisolone, but her symptoms did not improve. Due to concerns that the continued steroids would exacerbate her depressed mood, she continued to be treated with the symptomatic drugs duloxetine 20 mg, mecobalamin 1500 μg, and pregabalin 150 mg.

The primary lesion in the upper left lobe of her lung had shrunk, and it was no longer possible to measure its size because it was hidden by atelectasis. The lymph node metastasis also shrunk to the extent that it could not be detected on a CT scan. ProGRP was 36.2 pg/mL, which was within the normal range. Therefore, we believed that the patient’s lung cancer had maintained a complete response. Neurological symptoms, including sensory disturbance in the distal extremities and walking instability, remained stable, and her depressed mood and fatigue improved. Her PS score had risen to 2, and she was able to live her daily life with the help of others, including shopping. We decided to re-evaluate her condition, and we referred her to the Neurology department again for a detailed medical history and physical examination.

**Figure 1:**
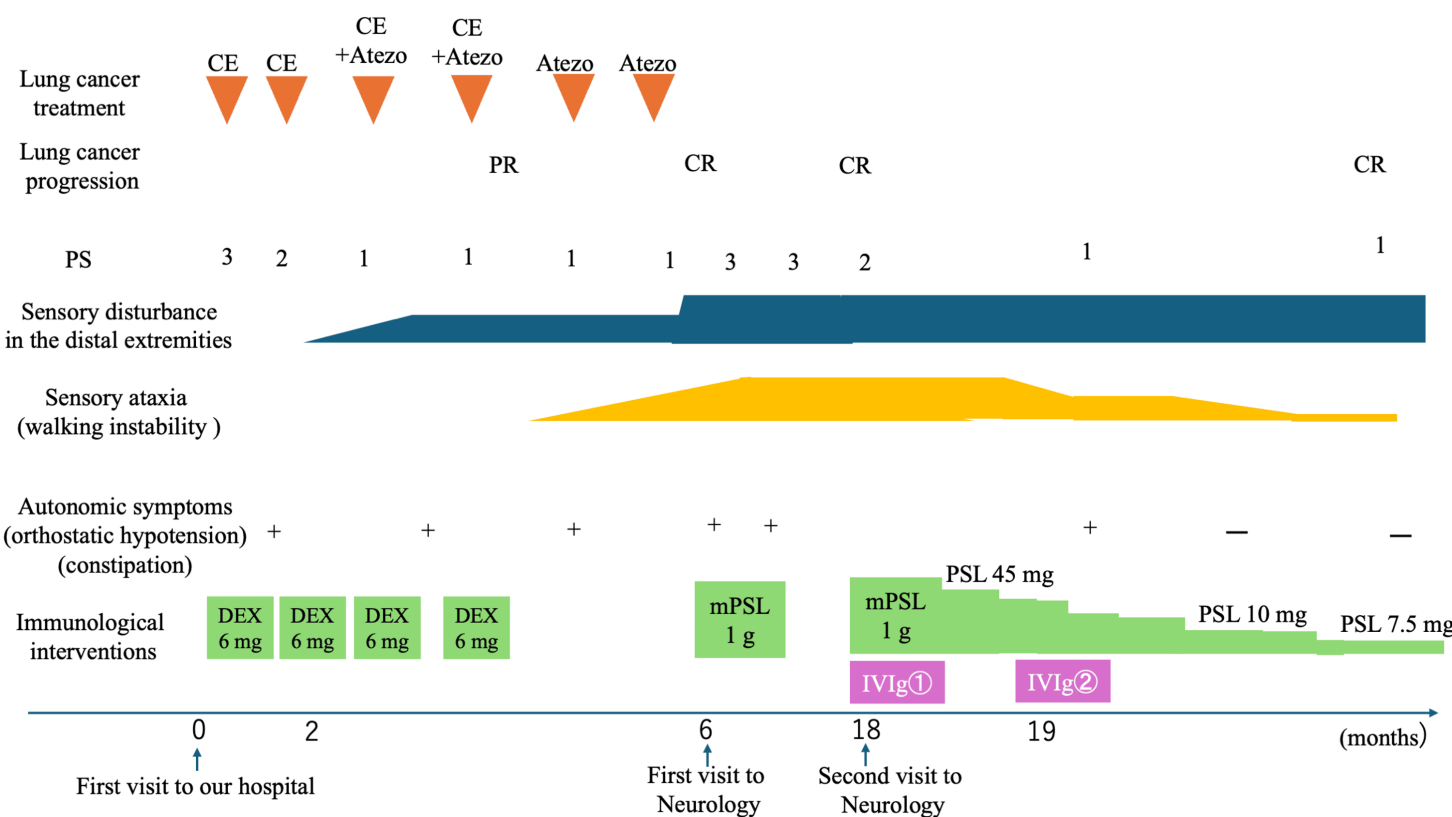
The clinical course of this case, lung cancer disease, treatment and patient performance status (PS), neurological symptoms, and immunological interventions are shown. Atezo: atezolizumab; CE: carboplatin and etoposide; CR: complete response; DEX: dexamethasone; IVIg: intravenous immunoglobulin therapy; mPSL: methylprednisolone; PR: partial response; PSL: prednisolone

**Figure 2:**
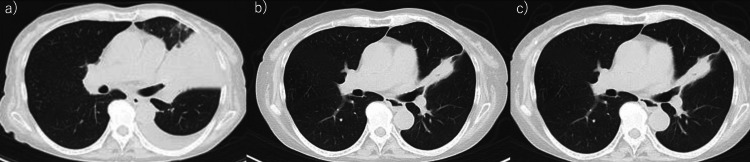
Computed tomography scans show the patient’s course during left upper lobe small-cell lung cancer. In her left upper lobe, the small-cell lung cancer mass continued to shrink after chemotherapy was discontinued. (a) First visit to our hospital. (b) First visit to the Neurology department. (c) Second visit to the Neurology department after treatment with steroids and intravenous immunoglobulin therapy.

At that time, there were no significant changes in physical findings, blood tests, serum antineural antibodies, nerve conduction tests, or brain MRI from her initial consultation at the Neurology department. She underwent a cerebrospinal fluid examination, which revealed no apparent abnormality or negative cerebrospinal fluid cytology. As her neurological symptoms had existed prior to the initiation of atezolizumab and had worsened after the initiation of atezolizumab, we considered that the neurological symptoms were due to PNS and worsened due to atezolizumab. Furthermore, her depressed mood and fatigue improved after levothyroxine replacement, partly due to hypothyroidism of irAEs involving the symptoms. In response to PNS, which had worsened due to atezolizumab, she received prednisolone after intravenous methylprednisolone. Furthermore, she received intravenous immunoglobulin(IVIg) in parallel with steroid treatment. Her symptoms of dysesthesia in the distal extremities persisted; however, her walking instability, orthostatic hypotension, and constipation improved. Over the next 2 years, her lung cancer was stable on CT, with no new metastases and no increase in the size of the primary lesion or lymph node metastases. In addition, her tumor markers remained within the normal range; therefore, we concluded that her lung cancer had not recurred. In addition, her neurological symptoms did not worsen (Figures 1-2).

## Discussion

PNS is a complication of various cancer types and is reported to occur in 1-9% of SCLCs [2,3]. A previous study revealed that 29% of PNS cases associated with SCLC developed after ICI administration [6]. The development of neurological symptoms may result in a decrease in PS, making it impossible to continue chemotherapy and decreasing the patient’s quality of life. PNS in SCLC is thought to be caused by immune-mediated mechanisms, and there have been reports of worsening PNS after the introduction of ICIs [8]. There have been several reports of PNS in SCLC positive for anti-Hu antibodies that worsened with ICIs. However, there have been no comprehensive reports, including those that were positive for other anti-neuronal antibodies. To date, 26 cases have been published in English and cataloged in the PubMed database [9-24]. There were also five cases of worsening neurological symptoms after the use of ICIs in patients who already had neurological symptoms prior to the administration of ICIs [12,13,15]; however, reviews of the clinical characteristics and safety of these cases are lacking.

The present case demonstrates the following two points: the clinical characteristics of various antineural antibody-positive PNS in SCLC exacerbated by ICIs and the clinical features and course of the use of ICIs in patients with neurological symptoms prior to treatment. PNS can present with various neurological symptoms that may decrease PS, which can decrease a patient’s quality of life (QoL) and interrupt treatment. In particular, since ICIs are now used more frequently in the treatment of SCLC, they are expected to be encountered more frequently than before. Healthcare professionals need to be aware of the relationship between the PNS of SCLC and ICIs.

It is additionally reported that antibodies are produced against neural tissues that share antigenicity with tumor antigens [23]. Immunologic therapies, including steroids, are generally used for PNS; however, the therapeutic response is frequently inadequate, especially in cases with antineural antibodies to intracellular antigens that do not improve neurologic symptoms, even after immunologic therapy. In the case of PNS caused by anti-neuronal antibodies that target intracellular antigens, it has been reported that neurological symptoms are unlikely to improve even after treatment with steroids or other immunotherapies because the nerves are often already irreversibly damaged by the beginning of treatment. In addition, it is thought that one of the reasons why immunotherapies such as IVIg are unlikely to be effective in the case of PNS caused by anti-neuronal antibodies that target intracellular antigens is that cytotoxic T lymphocytes are mainly involved in the pathogenesis of the disease [24]. In these cases, it is thought that cytotoxic T lymphocytes cause neurological dysfunction, which mechanism is thought as follows. Neoantigens resulting from mutations in the tumor genes encoding onconeural proteins are released upon apoptosis from tumors under attack by innate immune cells. These peptides are then processed and presented by antigen-presenting cells (APCs) to CD4+ helper T-cells via major histocompatibility complex class II (MHC II), which in turn will activate antigen-specific B cells into Ab-producing plasma cells, leading to the appearance of onconeural antibodies in serum and in CSF; however, additionally, CD8+ cytotoxic T lymphocytes via MHC class I molecules, which will cause the neurological dysfunction [25]. In recent years, chemotherapy for advanced SCLC has been increasingly combined with cytotoxic anticancer agents and ICIs, including atezolizumab. ICIs exert antitumor effects by inhibiting the PD-1/PD-L1 pathway and activating T-cells. Atezolizumab is thought to prevent T-cell inactivation by inhibiting PD-L1 expressed on tumor cells and APCs, maintaining and enhancing the antitumor immune response. In total, 26 cases of SCLC with PNS have been treated with ICIs [9-22]. Although the clinical characteristics of anti-Hu antibody-positive cases that worsened following ICIs treatment have been reported previously [9-19], a comprehensive review including PNS with positive antineural antibodies other than anti-Hu antibodies that worsened following ICIs treatment has not been reported [26]. We have summarized the clinical characteristics of 27 cases, including the present case, in Table 1 [9-22]. In these 27 cases, the overall age averaged 64.3 years (range 46-74), and the proportion of males was 74% (20/27). Atezolizumab was the most used ICI, at 63% (17/27), although the use of pembrolizumab, durvalumab, nivolumab, ipilimumab, and sintilimab was each reported in between one and three cases. In these, 88.9% of cases (24/27) were positive for anti-Hu antibodies, and several cases were positive for multiple antibodies. The most common neurological abnormality was sensory neuropathy (including subacute sensory neuropathy (SSN), polyneuropathy, painful paresthesia, sensory disturbance, and paralysis of the extremities) (44.4%, 12/27), and various neurological symptoms were observed, including dysarthria (18.5%, 5/27) and respiratory insufficiency (14.8%, 4/27). Steroids were used in most cases, whereas other immunosuppressive drugs, IVIg, and plasma exchange were used in some cases. In contrast, 37% of cases (10/27) reported improvement or partial improvement in neurological symptoms after treatment; 59.3% (16/27) reported no improvement, unchanged, or worsened symptoms; and 3.7% (1/27) reported unknown outcomes. Overall, atezolizumab was used more frequently, and many anti-Hu antibody-positive patients presented with peripheral sensory deficits and poor response to immunological therapy.

**Table 1:** Clinical characteristics, authors, and published literature of 27 cases of paraneoplastic neurological syndrome associated with small-cell lung cancer exacerbated by immune checkpoint inhibitors combined with previously reported cases and the present case. CY: Cyclophosphamide; GC: Glucocorticoids; IVIg: Intravenous immunoglobulin therapy; PE: Plasma exchange; SSN: Subacute sensory neuropathy *For those who had symptoms before ICI treatment, the period was summarized from the introduction of ICIs until the worsening of symptoms, and for those who had no symptoms before ICI treatment, the period was summarized from the introduction of ICIs until the appearance of symptoms.

	Age	Sex	ICI	Symptom	Antibody	Preceding symptoms (YES/NO)	Time to onset or progression^*^	Treatment	Treatment outcome	Author, year	Reference
1	46	M	Pembrolizumab	SSN	Hu	NO	After 4 courses	GC, IVIg	No change	Farina et al. 2023	[9]
2	70	M	Atezolizumab	Pancerebellar syndrome	Hu	NO	After 3 courses	GC, IVIg, CY, rituximab	No change	Farina et al. 2023	[9]
3	65	M	Durvalumab	Depression, memory deficits, SSN, flaccid tetraparesis, respiratory insufficiency	Hu	NO	After 4 courses	GC, PE, tocilizumab	No change	Farina et al. 2023	[9]
4	44	F	Atezolizumab	Temporal seizures, vertigo, memory deficits, dyscalculia, cerebellar ataxia	Hu	NO	After 5 courses	GC, IVIg	No change	Farina et al. 2023	[9]
5	56	M	Atezolizumab	Ophthalmoplegia, dysphagia, nystagmus, respiratory insufficiency, coma, dysautonomia	Hu	NO	After 1 course	GC, IVIg, CY, rituximab	Worsened	Farina et al. 2023	[9]
6	66	M	Atezolizumab	Memory impairment, new-onset seizure, hemichorea	Hu	NO	After 5 courses	NA	NA	Farina et al. 2023	[9]
7	57	M	Durvalumab	SSN, gastroparesis	Hu	NO	After 5 courses	GC	Worsened	Farina et al. 2023	[9]
8	76	M	Atezolizumab	SSN, nystagmus, diplopia, dysarthria, dysautonomia, respiratory insufficiency, coma	Hu	NO	After 2 courses	GC	Worsened	Farina et al. 2023	[9]
7	74	M	Atezolizumab	Memory deficits, altered behavior, hallucinations, progressing to akinetic mutism	Hu	NO	After 6 courses	GC	Worsened	Farina et al. 2023	[9]
10	64	F	Atezolizumab	Confusion, memory deficits, nystagmus, ataxia, dysphagia, respiratory insufficiency, coma	Hu	NO	After 3 courses	GC, IVIg	Worsened	Farina et al. 2023	[9]
11	62	F	Atezolizumab	limbic encephalitis, cerebellar ataxia, hearing loss, optic neuritis	Hu	NO	NA	GC, CY	Slightly improved	Sechi et al. 2020	[10]
12	70	M	Atezolizumab	SSN, sensory ataxia	Hu	NO	After 1 course	IVIg	No change	Morimoto et al. 2021	[11]
13	NA	M	Atezolizumab	Polyneuropathy, progressive numbness and weakness	Hu	YES	After 1 course	GC, PE	No improvement	Chompoopong et al. 2022	[12]
14	51	M	Nivolumab	SSN, ataxia	Hu	YES	After 3 courses	GC, IVIg, PE, CY	No improvement	Chompoopong et al. 2022	[12]
15	76	M	Atezolizumab	Memory loss, excitability	CRMP5, Hu, MA2, Ri, Tr, amphiphysin, Tr	YES	After 4 courses	GC	Improved	Tatsumi et al. 2020	[13]
16	70	M	Atezolizumab	Dizziness, sensory disturbance (tactile and pain)	Hu, SOX-1	NO	After 1 course	IVIg	No change	Morimoto et al. 2021	[11]
17	66	F	Sintilimab	Partial seizure	Hu	NO	After 1 course	GC	Improved	Kang et al. 2020	[14]
18	62	F	Nivolumab	Paralysis of extremities, dyskinesia, ataxia when walking	Hu	YES	After several weeks	GC, IVIg	No change	Raibagkar et al. 2020	[15]
19	71	F	Nivolumab + ipilimumab	Short-term memory impairment	Hu	NO	After 4 days	GC, natalizumab	Partly improved	Hottinger et al. 2018	[16]
20	46	M	Pembrolizumab	Painful paresthesia, gait disturbance	Hu	NO	After 3 months	GC, IVIg	Partly improved	Mongay-Ochoa et al. 2020	[17]
21	71	M	Atezolizumab	Dizziness, vomiting, diplopia, gait disturbance	Hu	NO	After 2 months	IVIg	Partly improved	Mongay-Ochoa et al. 2020	[17]
22	66	M	Atezolizumab	Impaired consciousness, dysphagia, gait disturbance	Hu	NO	After 2 months	GC, IVIg	Partly improved	Nakashima et al. 2022	[18]
23	69	M	Atezolizumab	Ocular clonus, myoclonus	Hu, SOX-1	NO	After 3 courses＋10 days	GC	No improvement	Arai et al. 2022	[19]
24	74	M	Atezolizumab	Muscle weakness, fatigue	Anti-P/Q-type voltage-gated calcium channel	NO	After 17 courses	GC, IVIg	Partly improved	Kunii et al. 2022	[20]
25	63	M	Ipilimumab	Nausea, vomiting, dysarthria	Yo, VGCC	NO	After 37 weeks	GC, infliximab	Worsened	Hardwick et al. 2022	[21]
26	70	M	Durvalumab	SSN, exhaustion	CV2, SOX-1, Zic4	NO	After 4 courses	GC, IVIg, PE	Improved	Wang et al. 2022	[22]
Present case	69	F	Atezolizumab	SSN, sensory ataxia, autonomic neuropathy	Hu, AMPH, Zic4, SOX-1, Ri	YES	After 2–4 courses	GC, IVIg	Partly improved	Present case	

The clinical characteristics, including the safety and therapeutic response of the introduction of ICIs in patients already presenting with PNS, have not been clarified. In a review of five cases, including the present case, the mean age was 64.5 (range 51-69) years, and the proportion of males was 60% (3/5). ICIs used were atezolizumab (60%, 3/5) and nivolumab (40%, 2/5). Antineural antibodies were positive for anti-Hu antibodies in 100% (5/5) and for multiple antibodies in 40% (2/5). The most common neurological abnormalities were sensory neuropathy (including SSN, polyneuropathy, and paralysis of extremities) (80%, 4/5), and memory loss (20%, 1/5). Steroids were administered in all cases. Cyclophosphamide, IVIg, and plasma exchange were administered in some cases. In contrast, 40% (2/5) reported an improvement or partial improvement in neurological symptoms after immunological treatment, and 60% (3/5) reported no improvement, change, or worsening. Although the number of cases collected was small, patients with SCLC neurological symptoms before ICI administration tended to present with worsening symptoms. These patients were less likely to have improved neurological symptoms with immunological intervention following ICI administration. In the present case, neurological symptoms worsened with the use of ICI for PNS in SCLC. At present, ICIs are widely used in cancers other than SCLC, and similar results have been reported in other types of cancer [27]. Central and peripheral neuropathies have also been reported in malignant melanoma after the introduction of ICIs [28]. In the present case, we describe the clinical characteristics of patients with various antineural antibody-positive PNS of SCLC who experienced worsening neurological symptoms after the administration of ICIs. The clinical characteristics and progression of ICI use in patients with pretreatment neurological symptoms are summarized in Table 2.

**Table 2:** Clinical characteristics, authors, and published literature of five previously reported cases of paraneoplastic neurological syndrome associated with small-cell lung cancer that worsened with immune checkpoint inhibitors, including this case and a case in which the patient had some neurological symptoms before receiving immune checkpoint inhibitors. CY: Cyclophosphamide; GC: Glucocorticoids; IVIg: Intravenous immunoglobulin therapy; PE: Plasma exchange; SSN: Subacute sensory neuropathy

	Age	Sex	ICI	Symptom	Antibody	Preceding symptoms (YES/NO)	Time to onset or progression	Treatment	Treatment outcome	Author, year	Reference
13	NA	M	Atezolizumab	Polyneuropathy, progressive numbness and weakness	Hu	YES	After 1 course	GC, PE	No improvement	Chompoopong et al. 2021	[12]
14	51	M	Nivolumab	SSN, ataxia	Hu	YES	After 3 courses	GC, IVIg, PE, CY	No improvement	Chompoopong et al. 2021	[12]
15	76	M	Atezolizumab	Memory loss, excitability	CRMP5, Hu, MA2, Ri, Tr, amphiphysin, Tr	YES	After 4 courses	GC	Improved	Tatsumi et al. 2020	[13]
18	62	F	Nivolumab	Paralysis of extremities, dyskinesia, ataxia when walking	Hu	YES	After several weeks	GC, IVIg	No change	Raibagkar et al. 2020	[15]
Present case	69	F	Atezolizumab	SSN, sensory ataxia, autonomic neuropathy	Hu, AMPH, Zic4, SOX-1, Ri	YES	After 2–4 courses	GC, IVIg	Partly improved	Present case	

Immunosuppressive drugs such as steroids are generally used for PNS; however they are frequently refractory to treatment. Several reasons for the lack of response to immunological therapy in paraneoplastic neuropathies have been assumed. Long-term exposure to T-cell-induced neuropathy causes irreversible changes in the nerves, resulting in an inadequate therapeutic response, even after the initiation of immunological treatment [28]. Peripheral sensory neuropathy was the most frequent symptom among the 27 patients. It was thought that the reason for peripheral sensory neuropathy symptoms is that Schwann cells in the peripheral nerves promote T-cell activation by presenting antigens and because the blood-nerve barrier in the peripheral sensory nerves is weak, allowing T-cells to invade easily [26]. Overall, 37% (10/27) of the patients described in Table 1 who presented with sensory peripheral neuropathy or sensory disturbance in the distal extremities showed improvement or partial improvement after immunological treatment. Only 2 out of 10 patients with sensory peripheral neuropathy or sensory disturbance in the distal extremities showed improvement or partial improvement after immunological treatment. In addition, one of these patients showed no improvement in peripheral sensory neuropathy after treatment. The poor response after immunological intervention in the five patients who already had neurological symptoms prior to the administration of ICIs may have caused irreversible neurological damage. However, cytotoxic anticancer drugs can also cause neurological symptoms, including peripheral neuropathy. The patient presented with sensory ataxia and autonomic neuropathy, which improved after immunological treatment. In contrast, her peripheral sensory deficits did not improve after immunological treatment. In other words, there was a mixture of positive (sensory ataxia and autonomic neuropathy) and negative (peripheral sensory neuropathy) responses to the immunological treatment. The reason for the order in which the neurological symptoms appeared, in this case, is unknown; however, since peripheral sensory disturbance was the first to appear, it may be that only the peripheral sensory disturbance had already become irreversible and did not improve even after treatment with steroids or IVIg. There was also a possibility that the peripheral sensory disturbance caused by the cytotoxic anticancer drugs (carboplatin and etoposide) also overlapped. To prevent such a situation, especially in SCLC, patients should be carefully examined daily for the presence of neurological symptoms, and PNS should be carefully investigated for its coexistence. If PNS is suspected, prompt therapeutic intervention and treatment options should be considered. As the therapeutic indications for ICIs are expected to expand in the future, the incidence of PNS is also expected to increase. To date, few cases have been reported, therefore, further case reports and the accumulation of knowledge are required. In this case, we determined that the series of neurological symptoms were due to PNS that had developed in conjunction with SCLC, worsened due to ICIs, and improved after immunological treatment. On the other hand, it is a limitation that we cannot rule out the possibility that the PNS itself had worsened or that carboplatin and etoposide had worsened the neurological symptoms.

## Conclusions

SCLC may also be accompanied by PNS, which can present with various neurological symptoms and can be exacerbated following ICIs. We believe that ICIs will be increasingly used in the future for SCLC and that we will encounter more cases of PNS that have merged with SCLC deteriorating after receiving ICIs. We suggest that it is important to pay attention to neurological symptoms or disturbances consistent with PNS because PNS can cause decreased PS, especially when introducing ICIs.
